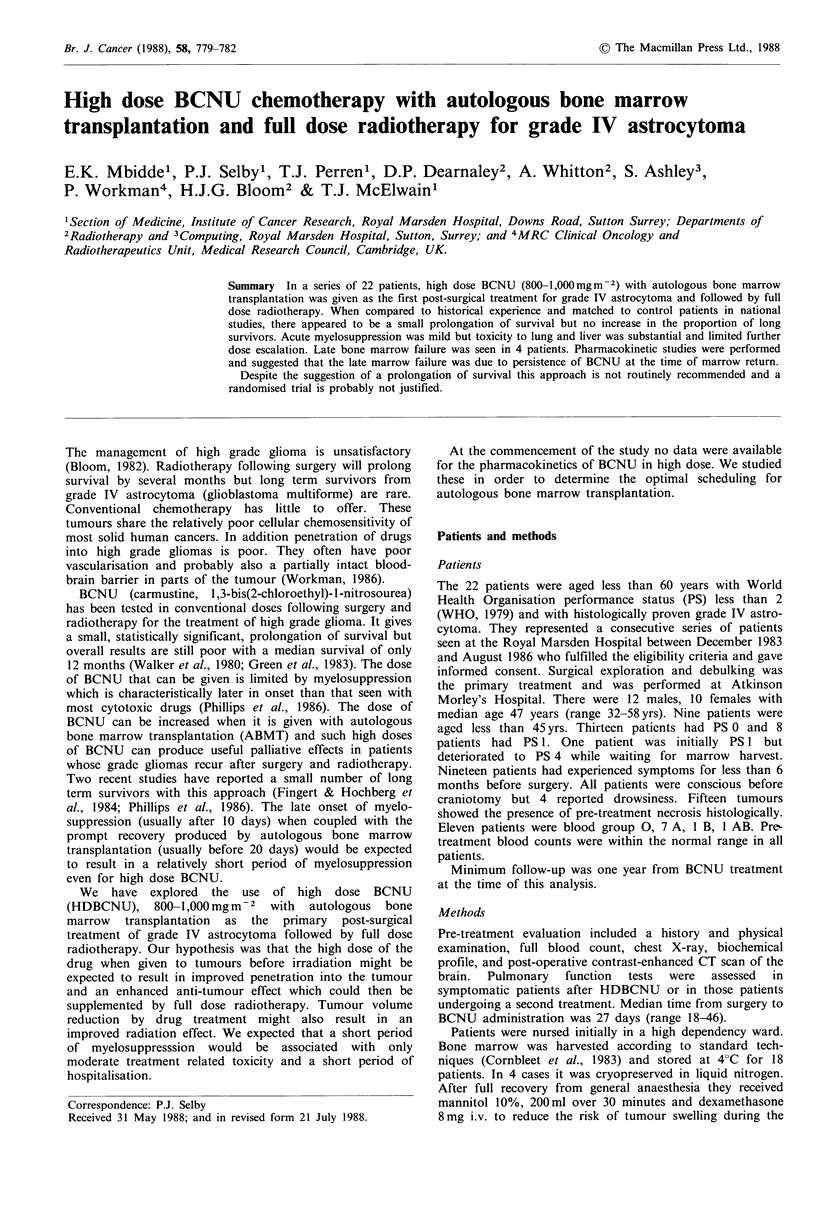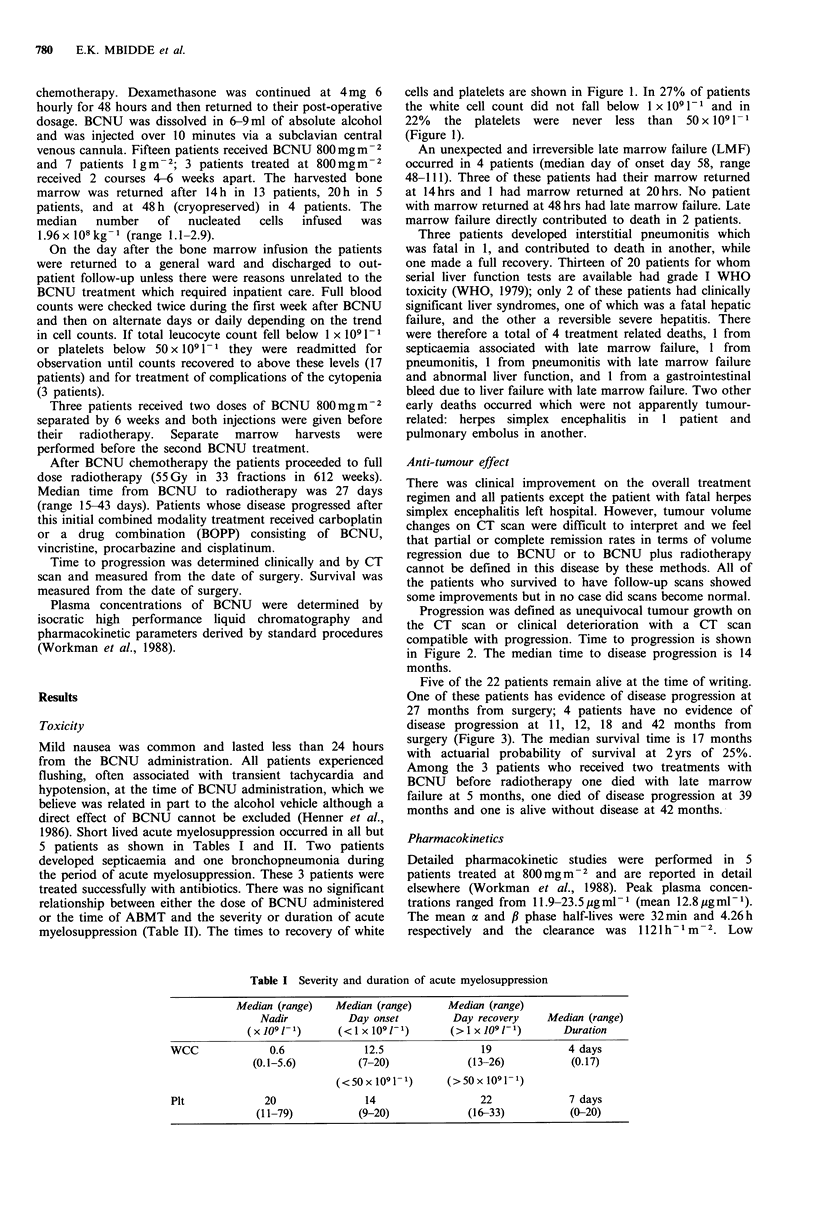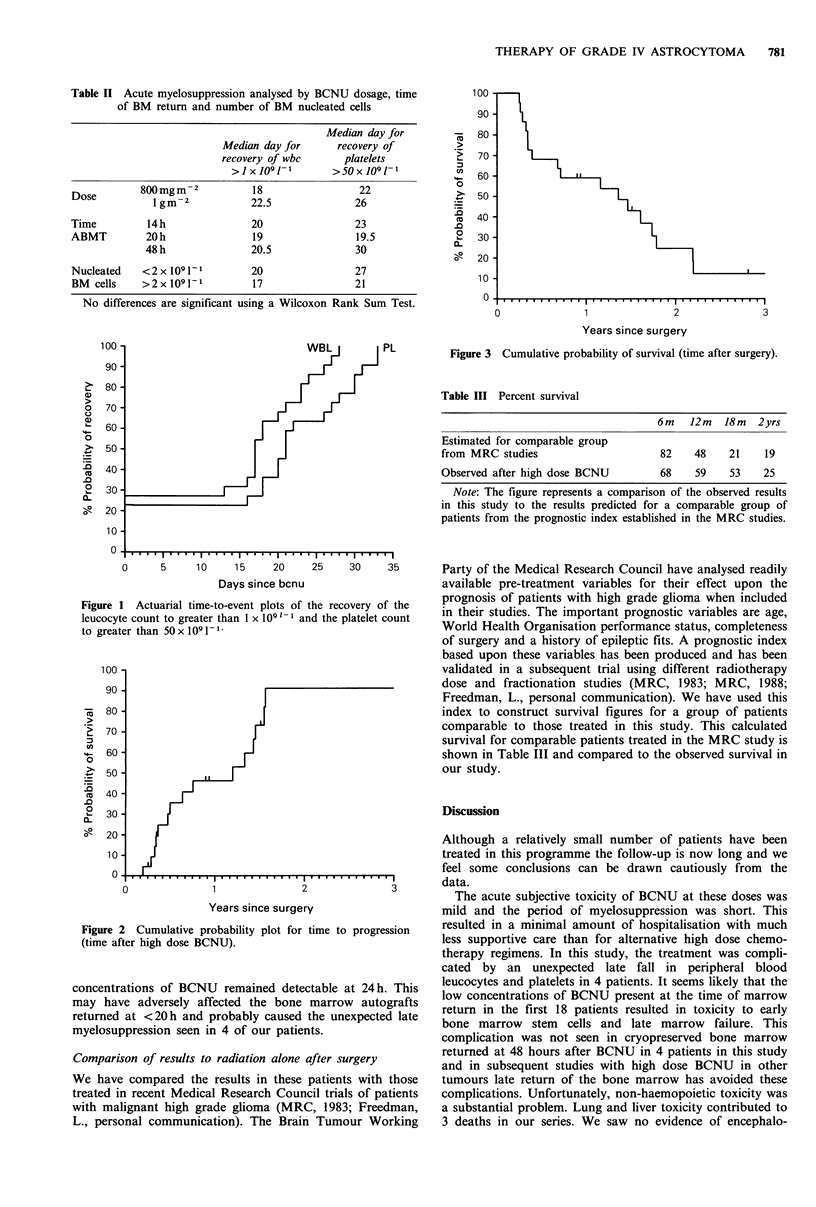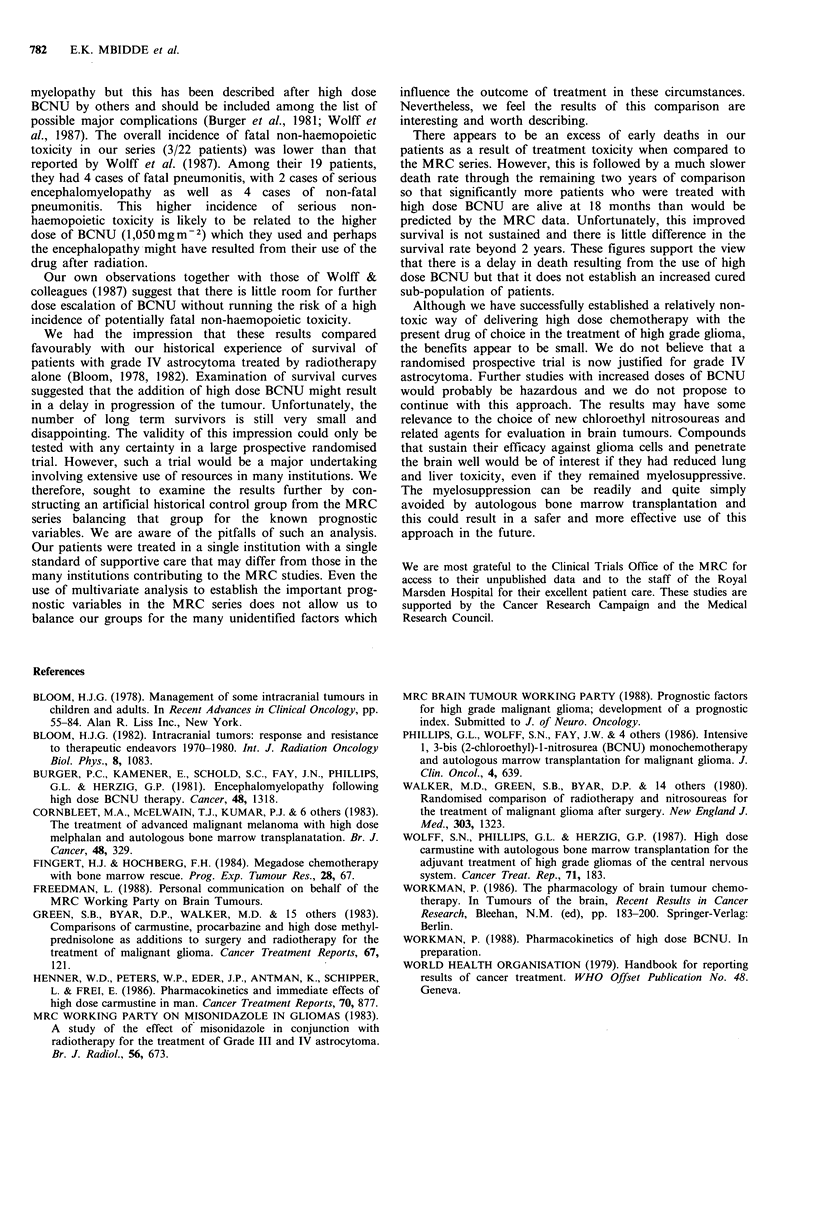# High dose BCNU chemotherapy with autologous bone marrow transplantation and full dose radiotherapy for grade IV astrocytoma.

**DOI:** 10.1038/bjc.1988.308

**Published:** 1988-12

**Authors:** E. K. Mbidde, P. J. Selby, T. J. Perren, D. P. Dearnaley, A. Whitton, S. Ashley, P. Workman, H. J. Bloom, T. J. McElwain

**Affiliations:** Section of Medicine, Institute of Cancer Research, Royal Marsden Hospital, Surrey, UK.

## Abstract

In a series of 22 patients, high dose BCNU (800-1,000mg m-2) with autologous bone marrow transplantation was given as the first post-surgical treatment for grade IV astrocytoma and followed by full dose radiotherapy. When compared to historical experience and matched to control patients in national studies, there appeared to be a small prolongation of survival but no increase in the proportion of long survivors. Acute myelosuppression was mild but toxicity to lung and liver was substantial and limited further dose escalation. Late bone marrow failure was seen in 4 patients. Pharmacokinetic studies were performed and suggested that the late marrow failure was due to persistence of BCNU at the time of marrow return. Despite the suggestion of a prolongation of survival this approach is not routinely recommended and a randomised trial is probably not justified.


Br. J. Cancer (1988), 58, 779–782 © The Macmillan Press Ltd., 1988

### High dose BCNU chemotherapy with autologous bone marrow transplantation and full dose radiotherapy for grade IV astrocytoma

E.K. Mbidde[1], P.J. Selby[1], T.J. Perren[1], D.P. Dearnaley[2], A. Whitton[2], S. Ashley[3], P. Workman[4], H.J.G. Bloom[2] & T.J. McElwain[1]

[1]*Section of Medicine, Institute of Cancer Research, Royal Marsden Hospital, Downs Road, Sutton Surrey; Departments of* [2]*Radiotherapy and* [3]*Computing, Royal Marsden Hospital, Sutton, Surrey; and* [4]*MRC Clinical Oncology and Radiotherapeutics Unit, Medical Research Council, Cambridge, UK.*

**Summary** In a series of 22 patients, high dose BCNU (800–1,000 $mg\,m^{-2}$) with autologous bone marrow transplantation was given as the first post-surgical treatment for grade IV astrocytoma and followed by full dose radiotherapy. When compared to historical experience and matched to control patients in national studies, there appeared to be a small prolongation of survival but no increase in the proportion of long survivors. Acute myelosuppression was mild but toxicity to lung and liver was substantial and limited further dose escalation. Late bone marrow failure was seen in 4 patients. Pharmacokinetic studies were performed and suggested that the late marrow failure was due to persistence of BCNU at the time of marrow return.

Despite the suggestion of a prolongation of survival this approach is not routinely recommended and a randomised trial is probably not justified.

The management of high grade glioma is unsatisfactory (Bloom, 1982). Radiotherapy following surgery will prolong survival by several months but long term survivors from grade IV astrocytoma (glioblastoma multiforme) are rare. Conventional chemotherapy has little to offer. These tumours share the relatively poor cellular chemosensitivity of most solid human cancers. In addition penetration of drugs into high grade gliomas is poor. They often have poor vascularisation and probably also a partially intact blood-brain barrier in parts of the tumour (Workman, 1986).

BCNU (carmustine, 1,3-bis(2-chloroethyl)-1-nitrosourea) has been tested in conventional doses following surgery and radiotherapy for the treatment of high grade glioma. It gives a small, statistically significant, prolongation of survival but overall results are still poor with a median survival of only 12 months (Walker *et al.*, 1980; Green *et al.*, 1983). The dose of BCNU that can be given is limited by myelosuppression which is characteristically later in onset than that seen with most cytotoxic drugs (Phillips *et al.*, 1986). The dose of BCNU can be increased when it is given with autologous bone marrow transplantation (ABMT) and such high doses of BCNU can produce useful palliative effects in patients whose grade gliomas recur after surgery and radiotherapy. Two recent studies have reported a small number of long term survivors with this approach (Fingert & Hochberg *et al.*, 1984; Phillips *et al.*, 1986). The late onset of myelosuppression (usually after 10 days) when coupled with the prompt recovery produced by autologous bone marrow transplantation (usually before 20 days) would be expected to result in a relatively short period of myelosuppression even for high dose BCNU.

We have explored the use of high dose BCNU (HDBCNU), 800–1,000 $mg\,m^{-2}$ with autologous bone marrow transplantation as the primary post-surgical treatment of grade IV astrocytoma followed by full dose radiotherapy. Our hypothesis was that the high dose of the drug when given to tumours before irradiation might be expected to result in improved penetration into the tumour and an enhanced anti-tumour effect which could then be supplemented by full dose radiotherapy. Tumour volume reduction by drug treatment might also result in an improved radiation effect. We expected that a short period of myelosuppresssion would be associated with only moderate treatment related toxicity and a short period of hospitalisation.

Correspondence: P.J. Selby
Received 31 May 1988; and in revised form 21 July 1988.

At the commencement of the study no data were available for the pharmacokinetics of BCNU in high dose. We studied these in order to determine the optimal scheduling for autologous bone marrow transplantation.

### Patients and methods

### *Patients*

The 22 patients were aged less than 60 years with World Health Organisation performance status (PS) less than 2 (WHO, 1979) and with histologically proven grade IV astrocytoma. They represented a consecutive series of patients seen at the Royal Marsden Hospital between December 1983 and August 1986 who fulfilled the eligibility criteria and gave informed consent. Surgical exploration and debulking was the primary treatment and was performed at Atkinson Morley's Hospital. There were 12 males, 10 females with median age 47 years (range 32–58 yrs). Nine patients were aged less than 45 yrs. Thirteen patients had PS 0 and 8 patients had PS 1. One patient was initially PS 1 but deteriorated to PS 4 while waiting for marrow harvest. Nineteen patients had experienced symptoms for less than 6 months before surgery. All patients were conscious before craniotomy but 4 reported drowsiness. Fifteen tumours showed the presence of pre-treatment necrosis histologically. Eleven patients were blood group O, 7 A, 1 B, 1 AB. Pre-treatment blood counts were within the normal range in all patients.

Minimum follow-up was one year from BCNU treatment at the time of this analysis.

### *Methods*

Pre-treatment evaluation included a history and physical examination, full blood count, chest X-ray, biochemical profile, and post-operative contrast-enhanced CT scan of the brain. Pulmonary function tests were assessed in symptomatic patients after HDBCNU or in those patients undergoing a second treatment. Median time from surgery to BCNU administration was 27 days (range 18–46).

Patients were nursed initially in a high dependency ward. Bone marrow was harvested according to standard techniques (Cornbleet *et al.*, 1983) and stored at 4°C for 18 patients. In 4 cases it was cryopreserved in liquid nitrogen. After full recovery from general anaesthesia they received mannitol 10%, 200 ml over 30 minutes and dexamethasone 8 mg i.v. to reduce the risk of tumour swelling during the chemotherapy. Dexamethasone was continued at 4 mg 6 hourly for 48 hours and then returned to their post-operative dosage. BCNU was dissolved in 6–9 ml of absolute alcohol and was injected over 10 minutes via a subclavian central venous cannula. Fifteen patients received BCNU 800 mg $m^{-2}$ and 7 patients 1 g $m^{-2}$; 3 patients treated at 800 mg $m^{-2}$ received 2 courses 4–6 weeks apart. The harvested bone marrow was returned after 14 h in 13 patients, 20 h in 5 patients, and at 48 h (cryopreserved) in 4 patients. The median number of nucleated cells infused was $1.96 \times 10^8$ $kg^{-1}$ (range 1.1–2.9).

On the day after the bone marrow infusion the patients were returned to a general ward and discharged to out-patient follow-up unless there were reasons unrelated to the BCNU treatment which required inpatient care. Full blood counts were checked twice during the first week after BCNU and then on alternate days or daily depending on the trend in cell counts. If total leucocyte count fell below $1 \times 10^9 l^{-1}$ or platelets below $50 \times 10^9 l^{-1}$ they were readmitted for observation until counts recovered to above these levels (17 patients) and for treatment of complications of the cytopenia (3 patients).

Three patients received two doses of BCNU 800 mg $m^{-2}$ separated by 6 weeks and both injections were given before their radiotherapy. Separate marrow harvests were performed before the second BCNU treatment.

After BCNU chemotherapy the patients proceeded to full dose radiotherapy (55 Gy in 33 fractions in 612 weeks). Median time from BCNU to radiotherapy was 27 days (range 15–43 days). Patients whose disease progressed after this initial combined modality treatment received carboplatin or a drug combination (BOPP) consisting of BCNU, vincristine, procarbazine and cisplatinum.

Time to progression was determined clinically and by CT scan and measured from the date of surgery. Survival was measured from the date of surgery.

Plasma concentrations of BCNU were determined by isocratic high performance liquid chromatography and pharmacokinetic parameters derived by standard procedures (Workman *et al.*, 1988).

### Results

### *Toxicity*

Mild nausea was common and lasted less than 24 hours from the BCNU administration. All patients experienced flushing, often associated with transient tachycardia and hypotension, at the time of BCNU administration, which we believe was related in part to the alcohol vehicle although a direct effect of BCNU cannot be excluded (Henner *et al.*, 1986). Short lived acute myelosuppression occurred in all but 5 patients as shown in Tables I and II. Two patients developed septicaemia and one bronchopneumonia during the period of acute myelosuppression. These 3 patients were treated successfully with antibiotics. There was no significant relationship between either the dose of BCNU administered or the time of ABMT and the severity or duration of acute myelosuppression (Table II). The times to recovery of white cells and platelets are shown in Figure 1. In 27% of patients the white cell count did not fall below $1 \times 10^9 l^{-1}$ and in 22% the platelets were never less than $50 \times 10^9 l^{-1}$ (Figure 1).

An unexpected and irreversible late marrow failure (LMF) occurred in 4 patients (median day of onset day 58, range 48–111). Three of these patients had their marrow returned at 14 hrs and 1 had marrow returned at 20 hrs. No patient with marrow returned at 48 hrs had late marrow failure. Late marrow failure directly contributed to death in 2 patients.

Three patients developed interstitial pneumonitis which was fatal in 1, and contributed to death in another, while one made a full recovery. Thirteen of 20 patients for whom serial liver function tests are available had grade I WHO toxicity (WHO, 1979); only 2 of these patients had clinically significant liver syndromes, one of which was a fatal hepatic failure, and the other a reversible severe hepatitis. There were therefore a total of 4 treatment related deaths, 1 from septicaemia associated with late marrow failure, 1 from pneumonitis, 1 from pneumonitis with late marrow failure and abnormal liver function, and 1 from a gastrointestinal bleed due to liver failure with late marrow failure. Two other early deaths occurred which were not apparently tumour-related: herpes simplex encephalitis in 1 patient and pulmonary embolus in another.

### *Anti-tumour effect*

There was clinical improvement on the overall treatment regimen and all patients except the patient with fatal herpes simplex encephalitis left hospital. However, tumour volume changes on CT scan were difficult to interpret and we feel that partial or complete remission rates in terms of volume regression due to BCNU or to BCNU plus radiotherapy cannot be defined in this disease by these methods. All of the patients who survived to have follow-up scans showed some improvements but in no case did scans become normal.

Progression was defined as unequivocal tumour growth on the CT scan or clinical deterioration with a CT scan compatible with progression. Time to progression is shown in Figure 2. The median time to disease progression is 14 months.

Five of the 22 patients remain alive at the time of writing. One of these patients has evidence of disease progression at 27 months from surgery; 4 patients have no evidence of disease progression at 11, 12, 18 and 42 months from surgery (Figure 3). The median survival time is 17 months with actuarial probability of survival at 2 yrs of 25%. Among the 3 patients who received two treatments with BCNU before radiotherapy one died with late marrow failure at 5 months, one died of disease progression at 39 months and one is alive without disease at 42 months.

### *Pharmacokinetics*

Detailed pharmacokinetic studies were performed in 5 patients treated at 800 mg $m^{-2}$ and are reported in detail elsewhere (Workman *et al.*, 1988). Peak plasma concentrations ranged from 11.9–23.5 $\mu g\, ml^{-1}$ (mean 12.8 $\mu g\, ml^{-1}$). The mean $\alpha$ and $\beta$ phase half-lives were 32 min and 4.26 h respectively and the clearance was 112 $l\, h^{-1} m^{-2}$. Low

**Table I** Severity and duration of acute myelosuppression

| | *Median (range)* *Nadir* ($\times 10^9 l^{-1}$) | *Median (range)* *Day onset* ($< 1 \times 10^9 l^{-1}$) | *Median (range)* *Day recovery* ($> 1 \times 10^9 l^{-1}$) | *Median (range)* *Duration* |
|---|---|---|---|---|
| WCC | 0.6 (0.1–5.6) | 12.5 (7–20) | 19 (13–26) | 4 days (0.17) |
| | | ($< 50 \times 10^9 l^{-1}$) | ($> 50 \times 10^9 l^{-1}$) | |
| Plt | 20 (11–79) | 14 (9–20) | 22 (16–33) | 7 days (0–20) |

**Table II** Acute myelosuppression analysed by BCNU dosage, time of BM return and number of BM nucleated cells

| | | *Median day for recovery of wbc* $>1\times10^9\,l^{-1}$ | *Median day for recovery of platelets* $>50\times10^9\,l^{-1}$ |
|---|---|---|---|
| Dose | 800 mg m$^{-2}$ | 18 | 22 |
| | 1 g m$^{-2}$ | 22.5 | 26 |
| Time | 14 h | 20 | 23 |
| ABMT | 20 h | 19 | 19.5 |
| | 48 h | 20.5 | 30 |
| Nucleated | $<2\times10^9\,l^{-1}$ | 20 | 27 |
| BM cells | $>2\times10^9\,l^{-1}$ | 17 | 21 |

No differences are significant using a Wilcoxon Rank Sum Test.

**Figure 1** Actuarial time-to-event plots of the recovery of the leucocyte count to greater than $1\times10^{9\,l^{-1}}$ and the platelet count to greater than $50\times10^9\,l^{-1}$.

**Figure 2** Cumulative probability plot for time to progression (time after high dose BCNU).

concentrations of BCNU remained detectable at 24 h. This may have adversely affected the bone marrow autografts returned at <20 h and probably caused the unexpected late myelosuppression seen in 4 of our patients.

*Comparison of results to radiation alone after surgery*

We have compared the results in these patients with those treated in recent Medical Research Council trials of patients with malignant high grade glioma (MRC, 1983; Freedman, L., personal communication). The Brain Tumour Working

**Figure 3** Cumulative probability of survival (time after surgery).

**Table III** Percent survival

| | *6 m* | *12 m* | *18 m* | *2 yrs* |
|---|---|---|---|---|
| Estimated for comparable group from MRC studies | 82 | 48 | 21 | 19 |
| Observed after high dose BCNU | 68 | 59 | 53 | 25 |

*Note*: The figure represents a comparison of the observed results in this study to the results predicted for a comparable group of patients from the prognostic index established in the MRC studies.

Party of the Medical Research Council have analysed readily available pre-treatment variables for their effect upon the prognosis of patients with high grade glioma when included in their studies. The important prognostic variables are age, World Health Organisation performance status, completeness of surgery and a history of epileptic fits. A prognostic index based upon these variables has been produced and has been validated in a subsequent trial using different radiotherapy dose and fractionation studies (MRC, 1983; MRC, 1988; Freedman, L., personal communication). We have used this index to construct survival figures for a group of patients comparable to those treated in this study. This calculated survival for comparable patients treated in the MRC study is shown in Table III and compared to the observed survival in our study.

### Discussion

Although a relatively small number of patients have been treated in this programme the follow-up is now long and we feel some conclusions can be drawn cautiously from the data.

The acute subjective toxicity of BCNU at these doses was mild and the period of myelosuppression was short. This resulted in a minimal amount of hospitalisation with much less supportive care than for alternative high dose chemotherapy regimens. In this study, the treatment was complicated by an unexpected late fall in peripheral blood leucocytes and platelets in 4 patients. It seems likely that the low concentrations of BCNU present at the time of marrow return in the first 18 patients resulted in toxicity to early bone marrow stem cells and late marrow failure. This complication was not seen in cryopreserved bone marrow returned at 48 hours after BCNU in 4 patients in this study and in subsequent studies with high dose BCNU in other tumours late return of the bone marrow has avoided these complications. Unfortunately, non-haemopoietic toxicity was a substantial problem. Lung and liver toxicity contributed to 3 deaths in our series. We saw no evidence of encephalomyelopathy but this has been described after high dose BCNU by others and should be included among the list of possible major complications (Burger *et al.*, 1981; Wolff *et al.*, 1987). The overall incidence of fatal non-haemopoietic toxicity in our series (3/22 patients) was lower than that reported by Wolff *et al.* (1987). Among their 19 patients, they had 4 cases of fatal pneumonitis, with 2 cases of serious encephalomyelopathy as well as 4 cases of non-fatal pneumonitis. This higher incidence of serious non-haemopoietic toxicity is likely to be related to the higher dose of BCNU (1,050 $mg\,m^{-2}$) which they used and perhaps the encephalopathy might have resulted from their use of the drug after radiation.

Our own observations together with those of Wolff & colleagues (1987) suggest that there is little room for further dose escalation of BCNU without running the risk of a high incidence of potentially fatal non-haemopoietic toxicity.

We had the impression that these results compared favourably with our historical experience of survival of patients with grade IV astrocytoma treated by radiotherapy alone (Bloom, 1978, 1982). Examination of survival curves suggested that the addition of high dose BCNU might result in a delay in progression of the tumour. Unfortunately, the number of long term survivors is still very small and disappointing. The validity of this impression could only be tested with any certainty in a large prospective randomised trial. However, such a trial would be a major undertaking involving extensive use of resources in many institutions. We therefore, sought to examine the results further by constructing an artificial historical control group from the MRC series balancing that group for the known prognostic variables. We are aware of the pitfalls of such an analysis. Our patients were treated in a single institution with a single standard of supportive care that may differ from those in the many institutions contributing to the MRC studies. Even the use of multivariate analysis to establish the important prognostic variables in the MRC series does not allow us to balance our groups for the many unidentified factors which influence the outcome of treatment in these circumstances. Nevertheless, we feel the results of this comparison are interesting and worth describing.

There appears to be an excess of early deaths in our patients as a result of treatment toxicity when compared to the MRC series. However, this is followed by a much slower death rate through the remaining two years of comparison so that significantly more patients who were treated with high dose BCNU are alive at 18 months than would be predicted by the MRC data. Unfortunately, this improved survival is not sustained and there is little difference in the survival rate beyond 2 years. These figures support the view that there is a delay in death resulting from the use of high dose BCNU but that it does not establish an increased cured sub-population of patients.

Although we have successfully established a relatively non-toxic way of delivering high dose chemotherapy with the present drug of choice in the treatment of high grade glioma, the benefits appear to be small. We do not believe that a randomised prospective trial is now justified for grade IV astrocytoma. Further studies with increased doses of BCNU would probably be hazardous and we do not propose to continue with this approach. The results may have some relevance to the choice of new chloroethyl nitrosoureas and related agents for evaluation in brain tumours. Compounds that sustain their efficacy against glioma cells and penetrate the brain well would be of interest if they had reduced lung and liver toxicity, even if they remained myelosuppressive. The myelosuppression can be readily and quite simply avoided by autologous bone marrow transplantation and this could result in a safer and more effective use of this approach in the future.

We are most grateful to the Clinical Trials Office of the MRC for access to their unpublished data and to the staff of the Royal Marsden Hospital for their excellent patient care. These studies are supported by the Cancer Research Campaign and the Medical Research Council.

### References

BLOOM, H.J.G. (1978). Management of some intracranial tumours in children and adults. In *Recent Advances in Clinical Oncology*, pp. 55–84. Alan R. Liss Inc., New York.

BLOOM, H.J.G. (1982). Intracranial tumors: response and resistance to therapeutic endeavors 1970–1980. *Int. J. Radiation Oncology Biol. Phys.*, **8**, 1083.

BURGER, P.C., KAMENER, E., SCHOLD, S.C., FAY, J.N., PHILLIPS, G.L. & HERZIG, G.P. (1981). Encephalomyelopathy following high dose BCNU therapy. *Cancer*, **48**, 1318.

CORNBLEET, M.A., McELWAIN, T.J., KUMAR, P.J. & 6 others (1983). The treatment of advanced malignant melanoma with high dose melphalan and autologous bone marrow transplanatation. *Br. J. Cancer*, **48**, 329.

FINGERT, H.J. & HOCHBERG, F.H. (1984). Megadose chemotherapy with bone marrow rescue. *Prog. Exp. Tumour Res.*, **28**, 67.

FREEDMAN, L. (1988). Personal communication on behalf of the MRC Working Party on Brain Tumours.

GREEN, S.B., BYAR, D.P., WALKER, M.D. & 15 others (1983). Comparisons of carmustine, procarbazine and high dose methylprednisolone as additions to surgery and radiotherapy for the treatment of malignant glioma. *Cancer Treatment Reports*, **67**, 121.

HENNER, W.D., PETERS, W.P., EDER, J.P., ANTMAN, K., SCHIPPER, L. & FREI, E. (1986). Pharmacokinetics and immediate effects of high dose carmustine in man. *Cancer Treatment Reports*, **70**, 877.

MRC WORKING PARTY ON MISONIDAZOLE IN GLIOMAS (1983). A study of the effect of misonidazole in conjunction with radiotherapy for the treatment of Grade III and IV astrocytoma. *Br. J. Radiol.*, **56**, 673.

MRC BRAIN TUMOUR WORKING PARTY (1988). Prognostic factors for high grade malignant glioma; development of a prognostic index. Submitted to *J. of Neuro. Oncology*.

PHILLIPS, G.L., WOLFF, S.N., FAY, J.W. & 4 others (1986). Intensive 1, 3-bis (2-chloroethyl)-1-nitrosurea (BCNU) monochemotherapy and autologous marrow transplantation for malignant glioma. *J. Clin. Oncol.*, **4**, 639.

WALKER, M.D., GREEN, S.B., BYAR, D.P. & 14 others (1980). Randomised comparison of radiotherapy and nitrosoureas for the treatment of malignant glioma after surgery. *New England J. Med.*, **303**, 1323.

WOLFF, S.N., PHILLIPS, G.L. & HERZIG, G.P. (1987). High dose carmustine with autologous bone marrow transplantation for the adjuvant treatment of high grade gliomas of the central nervous system. *Cancer Treat. Rep.*, **71**, 183.

WORKMAN, P. (1986). The pharmacology of brain tumour chemotherapy. In Tumours of the brain, *Recent Results in Cancer Research*, Bleehan, N.M. (ed), pp. 183–200. Springer-Verlag: Berlin.

WORKMAN, P. (1988). Pharmacokinetics of high dose BCNU. In preparation.

WORLD HEALTH ORGANISATION (1979). Handbook for reporting results of cancer treatment. *WHO Offset Publication No. 48*. Geneva.